# Predictive Value of Electromechanical Activation Time for In-Hospital Major Cardiac Adverse Events in Heart Failure Patients

**DOI:** 10.1155/2020/4532596

**Published:** 2020-01-02

**Authors:** Jing Zhang, Wen-Xian Liu, Shu-Zheng Lyu

**Affiliations:** ^1^Division of Cardiology, Coronary Care Unit, Beijing Anzhen Hospital, Capital Medical University, Beijing 100029, China; ^2^Division of Cardiology, Beijing Anzhen Hospital, Capital Medical University, Beijing 100029, China

## Abstract

**Objective:**

This prospective study aimed to evaluate the value of the cardiac cycle time-corrected electromechanical activation time (EMATc) measured at admission for predicting major cardiac adverse events (MACEs) in hospitalized patients with chronic heart failure (CHF).

**Methods:**

CHF patients with a left ventricular ejection fraction (LVEF) lower than 50% (*N* = 145) were enrolled in this study. Documented clinical end-points (MACEs) included cardiogenic death, onset of acute HF as assessed with invasive and noninvasive mechanical ventilation, and cardiogenic shock. According to the different clinical end-points, patients were divided into two groups: a MACE group (*n* = 22) and a nonMACE group (*n* = 123). EMATc, LVEF, and circulating levels of B type natriuretic peptide (BNP) and Troponin I (TnI) were measured. Multivariate logistic regression analysis was used to examine the association between EMATc and MACEs. The parameters adjusted in the multivariable model included EMATc, BNP, and heart rate. The predictive value of EMATc was evaluated by receiver operating characteristic (ROC) curve analysis.

**Results:**

Elevated EMATc was an independent risk factor for MACEs (*odds ratio [OR]* 1.1443, *95% confidence interval [CI]* 1.016–1.286, *P* = 0.027). The area under the ROC curve for EMATc was 0.799 (95% *CI* 0.702–0.896, *P* < 0.001). The optimal cutoff EMATc value was >13.8% with a sensitivity of 81.8% and a specificity of 65.9%.

**Conclusions:**

We demonstrated that an elevated EMATc measured at admission is an independent risk factor for MACEs among hospitalized CHF patients. Acoustic cardiography measured at admission may provide a simple, noninvasive method for risk stratification of CHF patients. This trial is registered with ChiCTR1900021470.

## 1. Introduction

Although advances in the diagnostic technologies and treatment of heart disease have been made over the past decade, the readmission and mortality rates among chronic heart failure (CHF) patients remain high. According to one report, the all-cause mortality of CHF patients was 17%, and the readmission rate within 12 months was 44% [1]. Hence, a more complete understanding of the risk factors for the occurrence of major adverse cardiac events (MACEs) associated with CHF is urgently needed [2]. The major cause of death for CHF patients is the aggravation of HF. Hence, a method for early assessment of the risk of developing MACEs as well as an early warning can significantly reduce both the mortality and medical cost associated with CHF [3, 4].

Echocardiography is a valuable tool for CHF diagnosis, as it noninvasively measures the structure of the heart chambers and the left ventricular ejection fraction (LVEF), and it is well-recognized that a decrease in the LVEF is associated with the severity of HF as well as a decrease in the survival rate [5]. However, echocardiography is expensive and time-consuming, and the results are affected by the skill and judgment of the medical technicians performing the examination. Therefore, other indexes, such as blood levels of B type natriuretic peptide (BNP), have been introduced. The European Society of Cardiology (ESC) guidelines for CHF recommend that a circulating BNP level lower than 100 pg/ml can exclude acute left HF [6, 7]. However, the plasma BNP level is affected by many factors, including atrial fibrillation, age, renal insufficiency, and body mass index (BMI) [8–10]. Therefore, the blood BNP level has limitations in its utility for HF prognosis. Thus, identification of novel parameters that are specific and convenient for predicting MACEs in CHF patients is needed, so that appropriate treatments can be initiated as early as possible.

Left ventricular (LV) systolic time intervals, including the preejection period (PEP), ejection time (ET), and their ratio (PEP/ET), are determined by the LV systolic and diastolic function and ventriculo-arterial coupling [11]. The electromechanical activation time (EMAT) is the interval from the beginning of electrical activation of LV (the onset of ECG Q wave) to the onset of the first sound (mitral valve closure) [12] and is one of the parameters that can be used to evaluate LV function by modern acoustic cardiography [13]. In general, the RR interval is used to standardize the EMAT, namely EMATc (EMAT/RRint), which represents a proportion of EMAT in the cardiac cycle. EMATc reflects the time required by LV systole to produce a sufficient pressure to close the mitral valve, while an increased EMATc is associated with a decline in LV systolic function in HF patients [3]. The objective of this study was to evaluate the correlation between EMATc and MACEs among in-hospital CHF patients and to examine the predictive value of EMATc for MACEs in these patients.

## 2. Methods

### 2.1. Study Population

A total of 145 patients who were treated for acute decompensation of CHF with a decreased LVEF between August 1, 2017 and December 31, 2018 were consecutively enrolled. HF patients with a LVEF <50% and New York Heart Association (NYHA) classification of III or IV were registered in this study. Patients who met one of the following criteria were excluded from this study: age <18 years, acute myocardial infarction, acute myocarditis, structural heart disease, acute episode of chronic obstructive pulmonary disease, plans for coronary intervention or cardiac surgery during this hospitalization, or the use of an angiotensin receptor-neprilysin inhibitor (ARNI). Informed consent was obtained from each participant, and the study was approved by the Institute Ethics Committee.

### 2.2. Data Collection

The demographic and baseline clinical data of all patients, including gender, age, medical history, and medications delivered and events that occurred during the course of the hospitalization, were recorded. The documented clinical data included resting heart rate (HR), blood pressure, and the levels of creatinine, BNP, and troponin I (TnI).

### 2.3. Acoustic Cardiography

Acoustic cardiography (AUDICOR, Inovise Medical, Inc., Portland, OR, USA) was performed after the patient had been admitted to the hospital for 2 hours and allowed to rest in a supine position for 5–10 minutes. Simultaneous electrocardiogram (ECG) and heart sound data from the V3/V4 standard precordial position were analyzed by the computerized algorithm to calculate the EMAT and the HR corrected variable EMATc. Three recordings were taken for each patient, and the average EMATc value was used in this study.

### 2.4. Echocardiography

The modified biplane Simpson's rule was used to measure LVEF. LV end diastolic volume (LVEDV) and LV end systolic volume (LVESV) were obtained from apical four- and two-chamber views (PHILIPS Affiniti 50). The investigator who interpreted the echocardiographic findings was blinded to all clinical data and acoustic cardiographic findings.

### 2.5. Endpoint

MACEs were defined as exacerbation of acute left HF that required invasive or noninvasive ventilator-assisted ventilation, cardiogenic shock, or cardiac death. Exacerbation of acute HF requiring invasive or noninvasive ventilator ventilation was referred to as the onset of dyspnea and/or severe hypoxemia during the course of standardized drug treatment (blood gas analysis of oxygen partial pressure <60 mmHg or peripheral oxygen saturation <90%). Cardiogenic shock generated from a cardiogenic cause was defined by a systolic pressure <90 mmHg for more than 30 min, or when a patient needed to be under the support of vasoactive drugs or intra-aortic balloon counter pulsation to maintain a blood pressure >90 mmHg or had pulmonary congestion when combined with symptomatic low perfusion of vital organs (e.g., persistent oliguria <30 ml/h or altered mental status).

### 2.6. Statistical Analysis

Continuous variables with a normal distribution were expressed as mean ± standard deviation (SD), whereas those variables with a skewed distribution were expressed as median with interquartile range [M (Q1, Q3)]. Two-sample Student's *t*-test or Mann Whitney *U* test were used for comparison of continuous variable data. Categorical variables were expressed as frequency (proportion), and the Chi-square test was used for data comparison between groups. Multivariate logistic regression analysis was used to analyze the independent risk factors of MACEs. Receiver operating characteristic (ROC) curves were used to analyze the predictive value of selected variables for MACEs among in-hospital HF patients. A two-sided *P* value <0.05 was considered statistically significant. All statistical analyses were performed using SPSS, version 22.0 (SPSS, Inc., Chicago, IL, USA).

## 3. Results

### 3.1. Comparison of Demographic and Baseline Clinical Characteristics of Patients with and Without MACEs

A total of 145 patients (106 males, 73.1%) with an average age of 57.9 ± 15.3 years were enrolled in this prospective study. These patients were divided into two groups according the different outcomes. The mean in-hospital duration was 7.50 (3.75, 10.25) days. The MACE group had 22 patients with MACEs, of whom 12 (54.5%) had exacerbation of acute left HF, 9 (40.9%) had cardiogenic death, and 1 had cardiogenic shock (4.5%). The nonMACE group two had 123 patients who did not experience any MACEs. There were no statistically significant differences with regard to age, gender, or prior medical history including hypertension, diabetes, myocardial infarction, percutaneous coronary intervention, coronary artery bypass grafting, or chronic renal insufficiency between the two groups (Table 1). HR and circulating BNP and TnI levels were significantly higher in the MACE group [*P* = 0.011, 0.004 and 0.010, respectively] than in the nonMACE group. However, the MACE group had a significantly lower LVEF (*P* = 0.006) but higher EMATc (*P* < 0.001) than the nonMACE group.

In the MACE group, the usage of a *β* receptor antagonist was significantly lower (*P* = 0.047; Table 2). There were no significant differences in the usage of angiotensin converting enzyme inhibitors/angiotensin receptor inhibitors (ACEIs/ARBs) and diuretics between the two groups.

### 3.2. Determination of the Predictive Value of BNP and EMATc for MACEs

**Figure 1:**
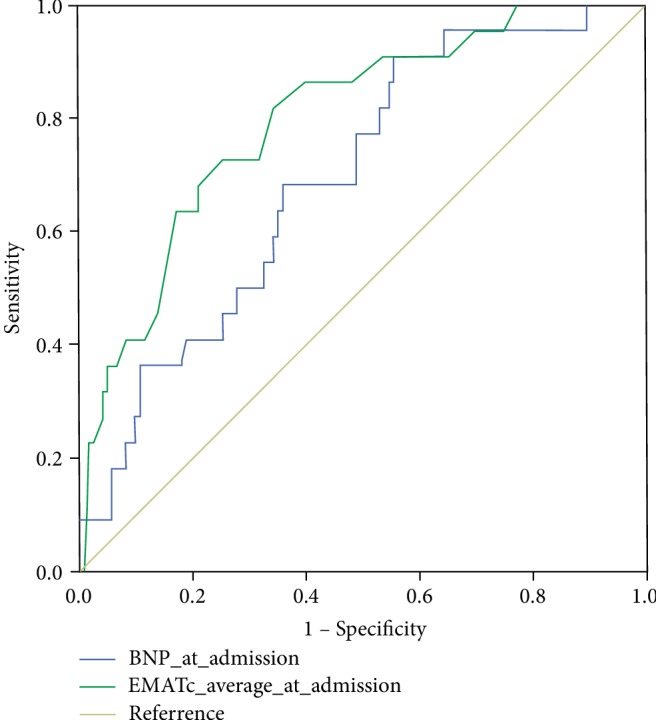
Determination of optimal cutoff points for EMATc and circulating BNP level. ROC curves were used to determine the cutoff points for eMATc and circulating BNP level. The green curve is for EMATc, and the AUC is 0.799 (95% *CI* 0.702–0.896,*P* < 0.001). The blue curve is for BNP, and the AUC is 0.700 (95% *CI* 0.602–0.799,*P* = 0.001).

We next performed ROC curve analyses to determine the value of BNP and EMATc for predicting MACEs in the hospitalized HF patients. As shown in Figure 1, the area under the ROC curve (AUC) for EMATc for the endpoint was 0.799 (95% *confidence interval [CI]* 0.702–0.896,*P* < 0.001), with an optimal cutoff point of 13.83%, a sensitivity of 81.8%, and a specificity of 65.9%. The AUC for BNP was 0.700 (95% *CI* 0.602–0.799, *P* = 0.001), with an optimal cutoff point of 811.5 pg/ml, a sensitivity of 67.7%, and a specificity of 65.8%.

### 3.3. Determination of Independent Risk Factors for MACEs

Clinical indicators that were analyzed for statistically significant differences (HR, TnI, BNP, LVEF% and EMATc) by univariate logistic regression are presented in Table 3. The parameters EMATc (*OR* 8.679, *95% CI* 2.760–27.291,*P* = 0.000), BNP (*OR* 3.714,* 95% CI* 1.409–9.791,*P* = 0.008), and HR (*OR* 1.032, *95% CI* 1.011–1.053,*P* = 0.003) were significantly different. There were no statistical differences in the parameters LVEF (*OR *3.667, *95% CI* 0.812–16.553,*P* = 0.091) and TnI (*OR* 3.082 *95% CI* 0.984–9.653,*P* = 0.053).

EMATc and BNP were transformed into categorical variables according to the cuttoff points, 13.8% and 811.5 pg/ml, respectively. TnI and LVEF were transformed into categorical variables according to the upper limits, 0.04 ng/ml and 40%, respectively.

Multivariate logistic regression analysis was used to identify independent risk factors for MACEs among in-hospital HF patients and showed that EMATc >13.8% (*OR* 6.578, *95% CI* 1.931–22.416,*P* = 0.003), and BNP >811.5 pg/ml (*OR* 3.398, *95% CI* 1.201–9.601,*P* = 0.021) were independent risk factors for MACEs (Table 4).

## 4. Discussion

In the present study, we identified EMATc as an independent risk factor for MACEs in in-hospital CHF patients and also identified the predicative value of EMATc for the occurrence of MACEs in these patients.

With the advances in the HF diagnosis and treatments, the survival rate of CHF patients has improved in the past 30 years, and the readmission rate has declined, but the clinical prognosis remains unappreciable [14]. The commonly used clinical measures for predicting these outcomes include LVEF and the plasma BNP level [15]. However, echocardiographic assessment of LVEF is expensive and requires skilled personnel. On the other hand, BNP plays an important role in excluding acute decompensated HF, and the elevation of circulating BNP levels suggests a poor prognosis in CHF patients [16]. However, BNP levels are influenced by a multitude of factors such as age, weight, and renal function, thus making interpretation complicated [14, 17]. Therefore, rapid, effective, and reliable clinical indicators are needed to screen HF patients for the risk of developing MACEs at the beginning of admission.

Previously, Carubelli et al. investigated the features of CHF patients with MACEs and found that LVEF was decreased significantly but the TnI level was increased [14]. Daniels and Maisel also reported that for every 100 pg/ml increase in BNP, the risk of mortality is elevated by 35% [18]. Consistent with the above findings, in the present study, we found that the BNP level was also elevated in the MACE group compared with that in nonMACE group.

Roos et al. [3]examined 37 HF patients undergoing cardiac catheterization using acoustic cardiography (17 with a LVEF <50%) and found that in patients with LV systolic dysfunction, EMAT was negatively correlated with LV dP/dt (*r* = −0.961,*P* = 0.063). Efstratiadis et al. [19] also evaluated cardiac function in 25 HF patients using LV catheterization, electrocardiography, and echocardiography and observed that systolic lengthening and LV pressure increased slowly when myocardial contractility decreased, while a prolonged EMAT was observed, suggesting delayed mechanical electrical coupling in HF patients. In addition to a lower LVEF, the patients with prolonged EMATc also had an increased LV systolic pressure, an elevated LV volume, a decreased ventricular systolic synchrony, and a diminished maximum LV dP/dt [19]. In line with the above findings, in the present study, we found that the MACE group had significantly prolonged EMATc, which coincided with an increased BNP level and decreased LVEF, compared with the nonMACE group. These observations indicate that the prolongation of EMATc was related to acutely decompensated HF.

Xu et al. [20] studied 128 HF patients with acoustic cardiography and 115 patients with normal cardiac function and found that EMAT >120 ms and EMATc >15% were associated with impaired LV systolic function. Moreover, they found that when EMATc was <10%, LV dysfunction could be ruled out. This conclusion was confirmed by Wang et al. who further found that EMATc was prolonged in HF patients with a LVEF <35% [21]. Collectively, these findings suggest that EMATc is a useful indicator of LV systolic function. In the present study, we demonstrated that EMATc was not only significantly different at baseline between the MACE and nonMACE groups, but also was an independent risk factor for in-hospital MACEs. The predictive value of EMATc for MACE was determined by ROC curve analysis (AUC 0.822 for a cutoff point of 13.88%, with a sensitivity of 86.4% and specificity of 68.2%). Thus, prolonged EMATc was associated with the risk of acute HF, cardiogenic shock, cardiac death and other adverse events. In addition, we believe that acoustic cardiography can be carried out bedside, is easy to perform, is repeatable and can be used to predict MACEs in CHF patients with a decreased LVEF.

Previously, Carubelli et al. found that TnI level and LVEF were independent risk factors affecting the long-term prognosis of HF patients. In our study, the differences in HR, BNP level, TnI level, and LVEF between the MACE and nonMACE groups were statistically significant. However, the above variables were not independent risk factors for in-hospital MACEs [14]. ESC guidelines recommend that a BNP <100 pg/ml [5]can be used to exclude acute left HF. In the present study, we showed that the risk of in-hospital MACEs increased with a higher level of BNP (AUC 0.700, *P* = 0.001for cutoff of 811.5 pg/ml), which was consistent with a previous report [19]. However, with this cutoff value, we did not find BNP to be an independent predictor for MACEs.

We noted that a previous study conducted by Chao et al. showed that approximately 45% patients had a normal ejection fraction [11]. Also, in their study, the outcome and survival rate were quite different between patients with HFrEF and those HFpEF. The patients enrolled in their study were diagnosed with acute heart failure syndrome, and it is possible that some bias may have been caused by the original diseases. We conducted the present study in order to determine the clinical significance of EMATc compared with the usual clinical parameters such as BNP, LVEF, and TNI. Therefore, our study had different objectives than theirs.

Some limitations of the present study need to be noted. First, we only studied the association of prolonged EMATc with MACEs in the hospitalized patients; future studies should be performed to examine the value of EMATs in the prognosis of HF patients over the long term. Second, we did not perform a long-term follow-up to examine the significance of EMATc in the management of patients with stable CHF. Third, this study was carried out in one institute with a small sample size. Fourth, the 2016 European Cardiology Association's HF guidelines redivided the classification of HF, classifying a LVEF level between 40% and 49% as intermediate zone HF (HFmrEF) [5]. We did not further analyze the predictive value of EMATs in the HFmrEF population. Fifth, our aim of this study was to analyze the clinical significance of EMATc. Thus, we focused only on the standard heart failure treatment. However, in our future research, we plan to include more treatments such as inotropic agents or vasodilators. Finally, this study took place from 2017 to 2018 when phase ARNI was not widely used widely in China. Therefore, we were not able to investigate the influence of ARNI on blood BNP levels.

## 5. Conclusions

In conclusion, we demonstrated that EMATc is an independent risk factor for MACEs among in-hospital HF patients and holds prognostic value. We believe that bedside acoustic cardiography detection of EMATc is simple, fast, and effective and should be used for screening hospitalized HF patients to stratify those more likely to suffer MACEs.

## Figures and Tables

**Table 1:** Comparison of demographic and baseline clinical characteristics between MACE and non-MACE groups.

	MACE (*n* = 22)	NonMACE (*n* = 123)	*P* value
Age (years)	55.9 ± 17.3	58.2 ± 15.0	0.506
Male	14 (63.6%)	92 (74.8%)	0.277

*Prior history*			
Hypertension	10 (45.5%）	69 (56.1%)	0.356
Diabetic mellitus	8 (36.4%)	38 (30.9%)	0.612
Prior myocardial infarction	4 (18.2%)	28 (22.8%)	0.784
Prior PCI or CABG	5 (22.7%)	12 (9.8%)	0.140
Chronic kidney disease	4 (18.2%)	20 (16.3%)	0.762

*Clinical characteristics*			
HR (bpm)	98 (81,122)	84 (72,98)	0.011
MBP (mmHg)	82.8 (66.2, 88.3)	87.7 (70.4, 101.0)	0.060
Cr (*μ*mol/L)	89.1 (76.0, 133.8)	85.7 (70.1, 104.8)	0.215
TnI (ng/ml)	1.11 (0.07, 10.07)	0.07 (0.02, 1.45)	0.010
BNP (pg/ml)	973 (626,1889)	618 (203,1052)	0.004
LVEF (%)	31.4 ± 8.3	36.7 ± 8.1	0.006
LVEDD (mm)	60.5 (55.7, 73.2)	58.0 (52.0, 64.0)	0.065
EMATc	16.5 (14.0, 20.2)	12.7 (10.3, 15.0)	<0.001

Data are presented as mean ± standard deviation for continuous variables with normal distribution, as median (interquartile range) for continuous variables with skewed distribution, and as frequency (percentage) for categorical variables.

**Table 2:** Comparison of treatments during hospitalization between MACE and nonMACE groups.

	MACE (*n* = 22)	NonMACE (*n* = 123)	*P* value
*β* receptor antagonist	10 (45.5%)	83 (67.5%)	0.047
ACEI/ARB	7 (31.8%)	65 (52.8%)	0.069
Diuretics	18 (81.8%)	89 (72.4%)	0.353

**Table 3:** Determination of risk factors for MACEs.

	*OR*	95% *CI*	*P*value
EMATc	8.679	2.760–27.291	0.000
LVEF (%)	3.667	0.812–16.553	0.091
TnI (ng/ml)	3.082	0.984–9.653	0.053
BNP (pg/ml)	3.714	1.409–9.791	0.008
HR (bpm)	1.032	1.011–1.053	0.003

**Table 4:** Determination of independent risk factors for MACEs.

	*OR*	95% *CI*	*P*value
EMATc	6.578	1.931–22.416	0.003
BNP (pg/ml)	3.398	1.201–9.601	0.021
HR (bpm)	1.015	0.992–1.038	0.196

## Data Availability

The datasets generated and analyzed during the present study are available from the corresponding author on reasonable request.
